# Policies and practices of SHEA Research Network hospitals during the COVID-19 pandemic

**DOI:** 10.1017/ice.2020.303

**Published:** 2020-06-23

**Authors:** Michael S. Calderwood, Valerie M. Deloney, Deverick J. Anderson, Vincent Chi-Chung Cheng, Shruti Gohil, Jennie H. Kwon, Lona Mody, Elizabeth Monsees, Valerie M. Vaughn, Timothy L. Wiemken, Matthew J. Ziegler, Eric Lofgren

**Affiliations:** 1 Dartmouth-Hitchcock Medical Center, Lebanon, New Hampshire; 2 Society for Healthcare Epidemiology of America, Arlington, Virginia; 3 Duke Center for Antimicrobial Stewardship and Infection Prevention, Durham, North Carolina; 4 Department of Microbiology, Queen Mary Hospital, Hong Kong Special Administrative Region, China; 5 Infection Control Team, Queen Mary Hospital, Hong Kong West Cluster, Hong Kong Special Administrative Region, China; 6 University of California, Irvine, California; 7 Washington University School of Medicine, St. Louis, Missouri; 8 University of Michigan and Veterans’ Affairs Ann Arbor Healthcare System, Ann Arbor, Michigan; 9 Children’s Mercy Kansas City, Kansas City, Missouri; 10 University of Michigan and Veterans’ Affairs Ann Arbor Healthcare System, Ann Arbor, Michigan; 11 Division of Infectious Diseases, Allergy, and Immunology, Saint Louis University Center for Health Outcomes Research/St Louis, Missouri; 12 University of Pennsylvania, Philadelphia, Pennsylvania; 13 Paul G. Allen School for Global Animal Health, Washington State University, Pullman, Washington

## Abstract

To understand hospital policies and practices as the COVID-19 pandemic accelerated, the Society for Healthcare Epidemiology of America (SHEA) conducted a survey through the SHEA Research Network (SRN). The survey assessed policies and practices around the optimization of personal protection equipment (PPE), testing, healthcare personnel policies, visitors of COVID-19 patients in relation to procedures, and types of patients. Overall, 69 individual healthcare facilities responded in the United States and internationally, for a 73% response rate.

In the past 40 years, healthcare epidemiologists have led responses to community and healthcare outbreaks of novel pathogens, including human immunodeficiency virus (HIV), severe acute respiratory syndrome coronavirus (SARS-CoV), Middle East respiratory syndrome coronavirus (MERS-CoV), novel influenza A virus (H1N1), and Ebola virus, in addition to the increased prevalence of antibiotic-resistant pathogens. Now they are handling novel coronavirus disease 2019 (COVID-19) pandemic responses in facilities across the world, adapting infection prevention and control practices rapidly to save lives while optimizing the use of supplies essential to patient care and healthcare worker safety.

The COVID-19 pandemic has presented a unique set of challenges, well beyond the rapid global spread of the severe acute respiratory syndrome coronavirus 2 (SARS-CoV-2). These unprecedented circumstances have demanded both careful thought as well as creativity by healthcare epidemiologists and infection prevention and control teams. While contending with the implications of presymptomatic and asymptomatic transmission^1^ among other uncertainties, the healthcare experience in the United States has been characterized by shortages of personal protective equipment (PPE), hand hygiene products, diagnostic tests, and test reagents, as well as breakdowns in the supply chain.^2,3^ These challenges have driven hospitals to enact unprecedented policies—from reprocessing disposable N95 respirators to the 3-dimensional printing of face shields.

Additionally, recommendations issued by public health authorities and professional societies have differed in key areas as the pandemic accelerated, particularly related to the type of respiratory protection to be worn in different settings and the indications for diagnostic testing. Facilities have had to rapidly identify and implement practices in the absence of unified guidance from authorities while providing reassurance to healthcare personnel (HCP) across sectors and roles when approaches differed from those issued by their trusted sources. In March and April 2020, professional societies continued to issue guidance and recommendations with the intent, in part, of helping their members advocate for access to the limited supply of PPE and diagnostic tests for procedures and scenarios.

The Society for Healthcare Epidemiology of America (SHEA) surveyed the SHEA Research Network (SRN) in April 2020 regarding some of these most challenging aspects of the pandemic. The findings show the “what,” but not the “why.” For example, the reasoning behind a facility’s decisions about precisely what PPE HCP should wear in specific circumstances may have been based on PPE stewardship, shortages, state and local laws, or other reasons. The results of the survey provide a “point-in-time” snapshot of a rapidly changing landscape and insight into how hospital epidemiologists from SRN member institutions have made swift adjustments during this crisis to help ensure patient and healthcare worker safety during the pandemic.

## Methods

With this SRN project, SHEA aimed to obtain an aggregated picture of common and novel approaches being taken by infection prevention teams to mitigate COVID-19 transmission risk during a time of extensive constraints and emerging scientific knowledge to understand how the pandemic was affecting facilities and to provide a resource to other institutions facing similar challenges. Conducted during April 2020, the survey asked hospital epidemiologists and infectious diseases specialists how they were adapting their facilities’ policies to match the growing body of evidence about SARS-CoV-2. The survey collected data pertaining to the challenges brought by product, equipment, and testing shortages. It was limited to 1 response per SRN facility; thus, responses represent the policies and practices of individual facilities, not individual people.

The SRN is a collaborative research consortium made up of 95 individual US and international healthcare facilities, each with a dedicated SRN principal investigator who oversees the facility’s response to research projects issued by the SRN. Overall, 91% of SRN facilities have infection control programs, with an average of 1.6 hospital epidemiologists and 5.3 infection preventionists. Typically, these facilities respond to 6–10 SRN research projects per year. Since the SRN was established in 2012, it has conducted nearly 50 research projects, and ~80% have been published in peer-reviewed literature.

In April 2020, SHEA created an electronic survey via the Survey Gizmo platform that was sent to SRN facilities on April 11, 15, 22, and 23, 2020. The week before it was launched, it was reviewed by current and former members of the SHEA Guidelines Committee and several authors of the Outbreak Response Training Program (ORTP) and the Prevention Course in Healthcare-Associated Infections Knowledge and Control (Prevention CHKC). The survey consisted of 21 questions and was estimated by the survey tool to require 17 minutes to complete (Appendix 1 online). It included a range of questions on current practices, and 5 matrices assessed PPE use for healthcare personnel (HCP), PPE optimization strategies, ethical considerations, visitor policies, and HCP policies in the context of use within the facility’s units, types of patients (eg, suspected or confirmed for COVID-19), and types of procedures.

The survey closed on April 23, 2020. Duplicates were removed, and the data were exported to Excel software (Microsoft, Redmond, WA) for analysis. SRN facilities are identified by an identification number (ID). If the ID appeared twice in the data export, these rows were assessed for completeness. Each facility has 1 SRN principal investigator who is responsible for obtaining the facility’s response; therefore, duplicate data rows usually indicate earlier incomplete response(s), and this was the case for this survey. Basic information about the SRN facilities was linked in aggregate to the survey findings using the SRN ID, and identifying information was removed.

The author panel was composed of SHEA Research Committee and Guidelines Committee members. Additionally, 2 consultants with expertise in data analysis and communications provided the first draft.

## Results

The survey (Appendix 1 online) had a response rate of 73% (69 of 95): 58 healthcare facilities from the United States and Canada and 11 facilities located internationally.

### Facility types

Of the responding hospitals, 57 had, through participation in the SRN, hospital-level information regarding the type and size of the hospital on file. Of these, 38 (67%) were academic medical centers or other teaching hospitals, 11 (19%) were community hospitals (8 with and 3 without an academic affiliation), and 7 (12%) were other types of hospitals, including Veterans’ Affairs hospitals, federal nonmilitary hospitals, and children’s hospitals. The total bed size was widely distributed (Table 1).

**Table 1.** SHEA Research Network (SRN) Facilities by Bed Size

Hospital Size, No. of Beds	No. (n=57)^a^	%
1–200	9	15.8
201–400	11	19.3
401–600	10	17.5
601–800	8	14.0
801–1,000	11	19.3
>1,000	7	12.3

a 1 unspecified.

### Geographic distribution

Most responses (78%) were from the United States, representing 30 states; 4 responses (6%) were from Canada; 3 were from Ontario; and 1 was from British Columbia. Outside North America, the SRN received responses from Brazil (n = 1), Egypt (n = 1), India (n = 2), Kenya (n = 1), Palestinian Territory (n = 1), the Republic of Korea (n = 1), Spain (n = 2), and Turkey (n = 2).

### COVID-19 patient prevalence

Of 66 respondents to this portion of the survey, 23 (35%) reported <5% of COVID-19 diagnostic tests being positive, 27 (41%) reported 6%–15% of tests being positive, 7 (11%) reported 16%–25% being positive, and 9 (14%) reported >25% being positive. Among hospitals in the United States and Canada, the average statewide or province-wide rate of reported COVID-19 cases was 48.2 cases per 10,000 people (standard deviation, 46.3). A heavily skewed distribution consisted of a few states or provinces experiencing severe outbreaks (eg, New York), whereas most localities had rates relatively close to the mean. The authors regressed the hospital-level test positivity rate identified by the respondent, with the statewide cumulative prevalence rate of confirmed COVID-19 cases (confirmed cases per state population). A 1-unit increase in the hospital COVID-19 test positivity rate (ie, from <5% to 6%–15%) was associated with an increased statewide COVID-19 rate of 28.32 cases per 10,000 people (95% confidence interval [CI], 18.39–38.24).^4,5^ The survey did not assess whether hospitals had experienced a surge of patients.

### Personal protective equipment

Respondents assessed the status of PPE in their facilities on a 5-point scale from “sustainable for pandemic” (score of 5) to “crisis level (almost out/none)” (score of 1). Averaged among all respondents, supply levels scored as follows: gloves (score, 4), eye coverings (3.7), surgical masks (3.6), respirators (3.5), and gowns (3.4). Of 60 facilities, 24 (40%) assessed their supply of respirators as being “limited” (expecting improvements or expecting declines) to “crisis level” (almost out/none), 9 (15%) assessed their supply as “sustainable for the pandemic,” and 27 (45%) assessed their supply as “adequate for the current situation.” The question did not distinguish among different types of respirators. Of the 60 facilities, 27 (45%) assessed their gown supplies as “limited” to “crisis level,” 9 (15%) assessed their gown supply as “sustainable for the pandemic,” and 24 (40%) assessed it as “adequate for the current situation.”

#### Universal masking

Approaches to the optimization of PPE use by HCP varied by settings of care and supply availability (Table 2). Of the 69 responding SRN facilities, 41 (59%) reported that their PPE strategies included universal respirator use by HCP in certain units, such as those dedicated to COVID-19 patients, the intensive care unit (ICU), or the emergency department (ED). Of the 69 responding institutions, 28 (41%) used surgical masks in all clinical care areas, including ambulatory areas, and 36 (~52%) used universal surgical masking throughout their entire facility. Higher levels of COVID-19 prevalence had a positive but not significant association with universal respirator use in facilities with higher COVID-19 test positivity rates (odds ratio [OR], 1.69; 95% CI, 0.94–3.04).

**Table 2.** Personal Protection Equipment (PPE) Optimization Strategies (n = 69)

Characteristic	Certain Units, No. (%)	All Clinical Care Areas (Including Ambulatory Areas), No. (%)	Entire Facility, No. (%)	Total Facilities, No. (%)
Universal HCP respirator use	41 (59.4)	0	0	41 (59.4)
Universal HCP surgical masking	5 (7.2)	28 (40.6)	36 (52.2)	63 (91.3)
**Extended Use**				
Respirator extended use, 1 d	26 (37.7)	6 (8.7)	5 (7.2)	36 (52.2)
Respirator extended use, >1 d	12 (17.4)	9 (13.0)	6 (8.7)	25 (36.2)
Respirator extended use with surgical mask or cloth mask	12 (17.4)	5 (7.2)	3 (4.3)	17 (24.6)
Gown extended use	7 (10.1)	6 (8.7)	6 (8.7)	19 (27.5)
**Reprocessing**				
Respirator reprocessing by vaporized hydrogen peroxide	11 (15.9)	7 (10.1)	8 (11.6)	23 (33.3)
Respirator reprocessing by ultraviolet irradiation	6 (8.7)	4 (5.8)	3 (4.3)	11 (15.9)
Respiratory reprocessing by ethylene oxide	1 (1.4)	1 (1.4)	1 (1.4)	3 (4.3)

Note. HCP, healthcare personnel.

Of the 69 facilities that responded to the survey, 43 (62%) had patients put on masks before HCP enter their rooms, and 25 of these 43 (58%) used this approach throughout the facility.

Among the 24 facilities that assessed their respirator supply as “limited” to “crisis-level,” 12 (50%) had universal HCP respirator use in place in certain units (eg, COVID-19, ICU, or ED), and no facilities reported universal HCP respirator use elsewhere in the facility. Among these same 24 facilities, 23 (96%) used universal HCP surgical masking, and 16 of these 23 facilities (70%) used this practice throughout the entire facility.

#### PPE optimization strategies

SRN facilities reported several strategies in response to limited supplies and availability of PPE (Table 2).

*1. Extended use*. The National Institute for Occupational Safety and Health (NIOSH) defines extended use as the practice of wearing the same N95 respirator for repeated close contact with several patients, without removal between patient encounters, in situations where multiple patients are infected with the same respiratory pathogen and patients are placed together.^6^ Of 69 responding facilities, 47 (68%) used 1 or more strategies involving the extended use of respirators. Having HCP in certain units wear the same respirator for 1 day was the most frequently cited strategy; it was utilized by 36 SRN facilities (52%). When not in use, 33 facilities (48%) reported that their HCP stored their respirators in a paper bag. This question did not distinguish among types of respirators.

Among the 24 facilities that assessed their respirator supply as “limited” to “crisis level,” 17 (71%) practiced some form of extended respirator use. Of the 27 facilities that assessed their gown supply as “limited” to “crisis level,” 16 (59%) practiced extended gown use or reuse.

*2. Self-producing.* In the “other” field in the question regarding self-producing test components, 9 of the 69 SRN respondents (13%) wrote in that they were self-producing PPE due to shortages, including face shields (n = 5), eye shields (n = 2), gowns (n = 1), surgical masks (n = 1), and coveralls (n = 2).

*3. Reprocessing.* Nearly half (n = 33) of the responding facilities indicated that they reprocessed respirators. Both the use of reprocessing and planning to do so had positive but not significant associations with increased COVID-19 test positivity rates (OR, 1.46; 95% CI, 0.84–2.52 and OR, 1.13; 95% CI, 0.66–1.94, respectively). Of the 69 respondents, 23 (33%) indicated that they were reprocessing respirators with vaporized hydrogen peroxide. Reprocessing with ultraviolet irradiation (n = 11) and ethylene oxide (n = 3) were less common. One respondent wrote in the “other” field that their facility used moist heat for 30 minutes to reprocess respirators.

#### Nasopharyngeal swabs

For HCP performing in-room collection of nasopharyngeal (NP) swab specimens from suspected or confirmed COVID-19 patients, 44 facilities (64%) recommended N95 respirators or the equivalent, identified in the survey as N95s, powered air purifying respirators (PAPRs), or half-mask respirators, which purify air with cartridges built into a rubber mask covering the nose and chin, based on “actual circumstances/supplies in the facility” (Table 3). Overall, 57 facilities (82%) recommended eyewear such as shields or goggles and 34 (49%) recommended a N95 respirator or equivalent with the eyewear. In addition, 24 facilities (35%) recommended a surgical mask with the eyewear, and 1 (1%) recommended eyewear (presumably a face shield) alone. The survey did not ask whether in-room nasopharyngeal swabs occurred in negative-pressure rooms. Also, 4 facilities (6%) used respirators for outdoor nasopharyngeal swab collection.

**Table 3.** Use of N95 or Equivalent Respirator by Procedure Type

Procedure	Suspected or Confirmed COVID-19 (n = 69), No. (%)				COVID-19 Not Suspected (n = 36), No. (%)			
	N95 or Equivalent	Surgical Mask	Eyewear	Respirator Allowed but Not Recommended	N95 or Equivalent	Surgical Mask	Eyewear	Respirator Allowed but Not Recommended
**Nasopharyngeal (NP) Swabs**								
In-room	44 (63.8)	25 (36.2)	57 (82.6)	6 (8.7)	11 (30.6)	11 (30.6)	15 (41.7)	3 (8.3)
Outdoor	40 (58.0)	25 (36.0)	55 (79.7)	5 (7.2)	11 (30.6)	10 (27.8)	15 (41.7)	2 (5.6)
**Upper and Lower Airway**								
Intubation	66 (95.6)	1 (1.4)	49 (71.0)	1 (1.4)	26 (72.2)	5 (13.9)	18 (50.0)	4 (11.1)
Bronchoscopy	66 (95.6)	2 (2.9)	48 (69.6)	0	25 (69.4)	3 (8.3)	17 (47.2)	4 (11.1)
Extubation	65 (94.2)	2 (2.9)	49 (71.0)	1 (1.4)	24 (66.7)	6 (16.7)	17 (47.2)	5 (13.9)
ENT surgery	63 (91.3)	4 (5.8)	48 (69.6)	1 (1.4)	22 (61.1)	5 (13.9)	16 (44.4)	3 (8.3)
Bag masking	62 (89.9)	4 (5.8)	49 (71.0)	1 (1.4)	20 (55.6)	8 (22.2)	16 (44.4)	4 (11.1)
ENT scope	62 (89.9)	1 (1.4)	48 (69.6)	0	20 (55.6)	6 (16.7)	16 (44.4)	3 (8.3)
HFOV	59 (85.5)	3 (4.3)	45 (65.2)	1 (1.4)	14 (38.9)	8 (22.2)	12 (33.3)	2 (5.6)
Induction of sputum	59 (85.5)	4 (5.8)	48 (69.6)	1 (1.4)	18 (50.0)	7 (10.1)	15 (41.7)	2 (5.6)
Medication via nebulizer	58 (84.1)	3 (4.3)	47 (68.1)	1 (1.4)	13 (36.1)	9 (13.0)	12 (33.3)	2 (5.6)
Noninvasive ventilation	57 (82.6)	7 (10.1)	46 (66.7)	1 (1.4)	13 (36.1)	10 (14.5)	13 (36.1)	2 (5.6)
HFNO with humidification	56 (81.2)	7 (10.1)	46 (66.7)	1 (1.4)	11 (30.6)	9 (13.0)	12 (33.3)	2 (5.6)
When ventilator circuits are broken	54 (78.3)	8 (11.6)	47 (68.1)	1 (1.4)	12 (33.3)	10 (14.5)	14 (38.9)	2 (5.6)
Chest compressions	54 (78.3)	7 (10.1)	46 (66.7)	1 (1.4)	11 (30.6)	13 (18.8)	16 (44.4)	3 (8.3)
Use of secretion-clearing devices	54 (78.3)	8 (11.6)	47 (68.1)	2 (2.9)	12 (33.3)	12 (17.4)	15 (41.7)	2 (5.6)
HFNO without humidification	53 (76.8)	7 (10.1)	47 (68.1)	1 (1.4)	11 (30.6)	9 (13.0)	11 (30.6)	2 (5.6)
Video-assisted thorascopic surgery	51 (73.9)	10 (14.5)	43 (62.3)	0	13 (36.1)	9 (13.0)	13 (36.1)	2 (5.6)
Presence of tracheostomy	46 (66.7)	13 (18.8)	50 (72.5)	4 (5.8)	10 (27.8)	13 (18.8)	14 (38.9)	1 (2.8)
Chest tube placement	37 (53.6)	22 (31.9)	46 (66.7)	4 (5.8)	8 (22.2)	13 (18.8)	12 (33.3)	2 (5.6)
**Obstetric and neonatal**								
Caesarean section	35 (50.7)	19 (27.5)	46 (66.7)	3 (4.3)	8 (22.2)	14 (38.9)	15 (41.7)	4 (11.1)
2nd stage of labor	32 (46.4)	19 (27.5)	45 (65.2)	2 (2.9)	8 (22.2)	13 (36.1)	15 (41.7)	4 (11.1)
Nonsurgical delivery	30 (43.5)	23 (33.3)	45 (65.2)	2 (2.9)	6 (16.7)	15 (41.7)	15 (41.7)	5 (13.9)
Care of preterm babies in isolates with NIV	39 (56.5)	15 (21.7)	45 (65.2)	1 (1.4)	8 (22.2)	13 (36.1)	12 (33.3)	3 (8.3)
**Cardiac**								
CABG with internal mammary artery	28 (40.6)	19 (27.5)	39 (56.5)	4 (5.8)	5 (13.9)	14 (38.9)	13 (36.1)	3 (8.3)
CABG with redo sternotomy	28 (40.6)	20 (29.0)	40 (58.0)	4 (5.8)	5 (13.9)	15 (41.7)	13 (36.1)	3 (8.3)
**Electrocautery**								
Electrocautery of blood	27 (39.1)	23 (33.3)	46 (66.7)	5 (7.2)	2 (5.6)	17 (47.2)	12 (33.3)	3 (8.3)
Electrocautery of GI tissue	30 (43.5)	22 (31.9)	47 (68.1)	5 (7.2)	3 (8.3)	17 (47.2)	13 (36.1)	3 (8.3)
Electrocautery of other body fluids	27 (39.1)	25 (36.2)	46 (66.7)	5 (7.2)	2 (5.6)	18 (50.0)	13 (36.1)	4 (11.1)
**Other**								
Endoscopy	46 (66.7)	15 (21.7)	45 (65.2)	4 (5.8)	13 (36.1)	12 (33.3)	16 (44.4)	5 (13.9)
Laparoscopy	32 (46.4)	25 (36.2)	46 (66.7)	3 (4.3)	7 (19.4)	16 (44.4)	14 (38.9)	5 (13.9)
Neurosurgery	34 (49.3)	26 (37.7)	47 (68.1)	4 (5.8)	5 (13.9)	16 (44.4)	14 (38.9)	5 (13.9)

Note. ENT, ear, nose, throat; HFOV, High-frequency oscillating ventilation; HFNO, High-flow nasal oxygen; CABG, coronary artery bypass grafting; NIV, noninvasive ventilation; GI, gastrointestinal.

#### Other procedures

Of 64 facilities, 36 (56%) recommended that HCP wear additional PPE for certain procedures on patients not suspected of COVID-19, that is, beyond the requirement for standard precautions (Table 3). These enhanced precautions had a positive but not significant association with higher test positivity rates (OR, 1.57; 95% CI, 0.87–2.81).

Moreover, 95% of SRN facilities, based on their current levels of supplies, recommended that HCP wear respirators (N95, PAPR, or half-mask respirator) for intubation, extubation, or bronchoscopy for confirmed or suspected COVID-19 patients. Approximately 90% of facilities recommended that HCP wear respirators for ear, nose, and throat (ENT) scope procedures and surgeries. More than 70% of SRN facilities recommended that HCP wear N95 respirators or the equivalent for the upper and lower airway procedures for suspected or confirmed COVID-19 patients. Moreover, 46 of the 69 SRN facilities (67%) recommended that HCP wear respirators for tracheostomy, and 37 (54%) recommended that HCP wear respirators for chest tube placement.

For patients not suspected of COVID-19, N95s or the equivalent were recommended for intubation by 26 of 36 SRN facilities (72%), for bronchoscopy by 25 of 36 SRN facilities 69%), for extubation by 24 of 36 SRN facilities (67%), for ENT surgery by 22 of 36 SRN facilities (61%), and for both bag masking and ENT scope by 20 of 36 SRN facilities (56%).

#### Testing availability

In total, 64 facilities responded to the questions regarding testing for COVID-19. Of these 64 facilities, 52 (81%) reported having access to in-house testing for COVID-19. Among 51 facilities that indicated the turnaround time for COVID-19 diagnostic test results, 22 (43%) reported <6 hours, 10% reported 7–12 hours, 10% reported 13–24 hours, and 18% reported >24 hours. Overall, 18 (26%) indicated that they self-produced test components: 13 (72%) self-produced viral transport media, 11 (61%) self-produced viral collection swabs, and 3 (16%) self-produced collection tubes due to shortages.

#### Diagnostic testing

Patients with respiratory symptoms were most commonly tested for COVID-19 in 65 of 66 facilities (98%), followed by those with isolated fever, who were tested in 57 of 66 facilities (86%). Those with gastrointestinal (GI) symptoms were tested in 40 of 66 facilities (61%), and asymptomatic patients undergoing certain procedures were tested in 42 of 66 facilities (64%). At the time of the survey, the Centers for Disease Control and Prevention (CDC) recommended testing of patients with symptoms according to 3 priority categories.^7^ The CDC defined symptoms of COVID-19 as “fever, cough, shortness of breath,” which it expanded on April 17, 2020, to also include “difficulty breathing, chills, repeated shaking with chills, muscle pain, headache, sore throat, and new loss of taste or smell”^8^ (Appendix 2 online). The survey was conducted before the publication of the *Infectious Diseases Society of America Guidelines on the Diagnosis of COVID-19*, which conditionally recommends testing of asymptomatic individuals before major time-sensitive surgeries.^9^

#### Preprocedural testing

Of 66 facilities, 42 (64%) reported testing hospitalized (n = 41) and nonhospitalized (n = 16) asymptomatic patients prior to certain procedures (Table 4). Respondents wrote in the procedures for which their facility tested asymptomatic patients; therefore, the procedures listed in Table 4 are categorized into common types but were not defined in the survey. Procedures specified by respondents are noted in the right column of Table 4.

**Table 4.** Preprocedural Testing on Asymptomatic Patients (n = 42)

Procedure Type	Facilities Testing, No. (%)	Specific Examples Provided by Respondents
All procedures	9 (21.4)	With exception of radiology for one response
Upper airway	9 (21.4)	ENT, tracheostomy, bronchoscopy, intubation
Labor and delivery	6 (14.3)	Including vaginal, Cesarean section, scheduled
Endoscopy	5 (11.9)	Upper endoscopy, colonoscopy, ERCP, EUS, laparoscopy, peritoneal insufflation, GI endoscopy
Lower airway	5 (11.9)	Intrathoracic, interventional pulmonary, bronchoscopy, pulmonary
Aerosol-generating procedures	4 (9.5)	Specifically written by respondent as “aerosol-generating procedure” by respondent
Sedation	4 (9.5)	General endotracheal anesthesia
Multiple procedures (unspecified)	4 (9.5)	
All admissions	3 (7.1)	
Cardiac	3 (7.1)	Electrophysiology, cardiac catheter lab, pacemaker, transesophageal echo test
Chemotherapy	3 (7.1)	Lymphodepleting therapy
Dental/facial	2 (4.8)	Oral and maxillofacial surgery
Elective surgery	2 (4.8)	Including if surgery could be postponed or clinical treatment altered based on positive test
Gastrointestinal	2 (4.8)	Intra-abdominal
Transplant	2 (4.8)	Allograft, pretransplant, autologous stem cell transplant
Emergency surgery	1 (2.4)	Within 48 h
Neurologic	1 (2.4)	Electroconvulsive therapy

Note. ENT, ear, nose, and throat; ERCP, endoscopic retrograde cholangiopancreatography; EUS, endoscopic ultrasound.

**Table 5.** Visitors of Patients Suspected or Confirmed for COVID-19

Characteristic	Facilities, No./Total (%)	Visitors, No. (No./Total, %)	PPE Required, No./Total (%)
Visitors not allowed for COVID-19 patients	15/64 (23.4)	N/A	N/A
Some or all visitors allowed	49/64 (76.6)	N/A	N/A
Visitors allowed for all COVID-19 patients	5/64 (7.8)	N/A	N/A
End of life	45/64 (70.3)	1 (31/45, 68.9) 2 (17/45, 37.8) 3 (1/45, 2.2) 1+, depending (4/45, 8.9)	41/45 (91.1)
Birthing partners^a^	37/63 (58.7)	1 (36/37, 97.3) 2 (1/37, 2.7)	28/37 (75.7)
Pediatric patients^a^	40/63 (63.4)	1 (34/40, 85) 2 (6/40, 15)	31/40 (77.5)

a One facility opted out of the question regarding birthing partners and pediatric patients.

#### Testing before discharge

Of 66 facilities, 32 (49%) tested hospitalized (n = 30) and nonhospitalized (n = 9) asymptomatic patients prior to discharge to skilled nursing facilities and long-term acute-care hospitals. Also, 22 of these 66 facilities (33%) tested hospitalized asymptomatic patients prior to discharge to outpatient hemodialysis units. Testing prior to discharge was associated with increased COVID-19 test positivity rates for discharge to outpatient hemodialysis units (OR, 1.92; 95% CI, 1.07–3.45) but not to skilled nursing facilities (OR, 1.36; 95% CI, 0.79–2.34).

#### Serologic testing

Of 64 facilities, 35 (55%) reported that they did not know whether they would consider a positive immunoglobulin G (IgG) result indicative of COVID-19 immunity. Of these 64 facilities, 22 (34%) responded that they would consider a positive serologic test indicative of immunity, 4 (6%) indicated that they would not consider it indicative of immunity, and 3 (5%) responded “other” (ie, if neutralizing antibody titers are available, if knowledge improves the correlation of a positive test with immunity, or in combination with a polymerase chain reaction [PCR] test to confirm infectivity). Facilities with higher COVID-19 test positivity rates were more likely to consider a positive serologic test as evidence for immunity (compared to “don’t know,” “no,” or “other”), though this association was not significant (OR, 1.23; 95% CI, 0.70–2.18).

### Visitors

Of the 64 facilities that completed this section of the survey, 5 (8%) allowed visitors for patients with suspected or confirmed COVID-19 in all circumstances, 15 (23%) did not allow visitors, and 44 (69%) allowed visitors in certain circumstances (Table 5). Of these 64 facilities, 45 (70%) allowed end-of-life visits, and 4 (9%) were flexible in the number of visitors (eg, immediate family was allowed >1 visitor if 1 person at a time, or as determined on a case-by-case basis) (Table 5). Of all 37 facilities that allowed birthing partners, 36 (97%) permitted 1 visitor per patient. Moreover, 34 of 40 facilities (85%) allowed 1 visitor to a pediatric patient, in most cases the parent; 6 of 40 facilities (15%) allowed 2 visitors for minors. Among respondents that allowed visitors of suspected or confirmed COVID-19 patients, 41 of 45 facilities (91%) required visitors to wear PPE, especially during end-of-life visits (Table 5). Facilities with higher positive COVID-19 test rates were more likely to expressly prohibit visitors for patients with suspected or confirmed COVID-19, though this association was not significant (OR, 1.54; 95% CI, 0.82–2.88).

### Healthcare personnel policies

#### Symptom screening of healthcare personnel

Of the 69 responding SRN facilities, 45 (65%) reported that they were doing HCP symptom checks once daily and 7 (10%) reported checking symptoms twice daily. COVID-19 test positivity rates were positively but not significantly associated with the absence of a symptom screening policy for HCP (OR, 1.28; 95% CI, 0.52–2.03). Two facilities reported that they were conducting daily PCR testing of HCP, and 44 (64%) reported doing contact tracing for COVID-19–positive healthcare personnel. Of these 44 facilities, 31 (70%) did this for all COVID-19–positive HCP in the facility.

#### Return to work

Of the 66 facilities that completed this part of the survey, 34 (52%) reported following the CDC non–test-based return-to-work criteria.^10^ The survey was conducted during a time when the CDC updated its return to work guidance for healthcare personnel, adding a preference for the use of the test-based strategy.^10^ Of these 66 facilities, 15 (23%) followed the CDC test-based return-to-work criteria, 21 (32%) used crisis-level mitigation strategies with evaluation by the occupational health department, and 3 (5%) used crisis-level mitigation strategies with certain restrictions. Also, 6 of these 66 facilities (9%) reported using >1 strategy.

#### Scrubs, laundering, and onsite accommodations

Of 69 responding facilities, 37 (54%) indicated that they provided scrubs for HCP; 32 (46%) had facility laundering of scrubs at the end of shifts, and 23 (33%) offered an onsite shower for HCP at the end of shifts. Also, 25 facilities (36%) provided onsite or local accommodations during the pandemic, and 17 (68%) of these accommodations being for HCP working in COVID-19, ICU, or ED units. These policies often co-occurred. For example, of the 38 facilities reporting the availability of laundry, showers, or onsite accommodations, 25 of these 38 (66%) provided >1. Higher COVID-19 test positivity rates were positively but not significantly associated with these 3 policies: showers (OR, 1.33; 95% CI, 0.69–2.50), laundry (OR, 1.22; 95% CI, 0.63–2.35) and lodging (OR, 1.33; 95% CI, 0.70–2.54).

### Ethical guidance sought for clinical or policy decisions

Facilities reported receiving ethical guidance, primarily from their institutions. Of the 69 responding facilities, 44 (64%) received guidance on potential COVID-19 therapeutics, 46 (67%) received guidance on PPE optimization strategies, 42 (61%) received guidance on patient triage, 35 (51%) received guidance on modifications, and 44 (64%) received guidance on visitor policies. States provided ethical guidance related to PPE optimization to 23 facilities (33%), states provided ethical guidance related to visitor policies to 23 facilities (33%), and states provided ethical guidance related to patient triage to 21 facilities (30%). Ethical guidance issued by professional organizations focused on potential COVID-19 therapeutics in 21 facilities (30%), on PPE optimization in 22 facilities (32%), and on patient triage in 16 facilities (23%). Of the topics in the survey for which facilities reported having received ethical guidance, PPE optimization was the most common from institutions, states, and professional organizations. Among all 69 respondents, 6 (9%) reported seeking guidance on equipment modifications, 5 (7% reported seeking guidance on therapeutics, and 5 (7%) reported seeking guidance on PPE optimization.

## Discussion

The findings of this survey illustrate how healthcare epidemiologists and infection control programs have adapted practices and policies in real time in response to emerging evidence, limited supplies, and divergent recommendations by public health authorities and professional organizations in essential frontline infection prevention practices during the COVID-19 pandemic. The data show areas of consistency, for example, utilization of N95 respirators (or equivalent) for upper and lower airway surgeries, as well as areas in which practices varied (eg, types of PPE used by HCP for nasopharyngeal swabbing). Variation in the type of isolation precautions used for certain procedures during the COVID-19 pandemic was higher than expected among healthcare facilities dealing with other viral respiratory pathogens. This finding provides insight into the complexity faced by healthcare facilities as they worked to synthesize rapidly emerging scientific information while managing turbulent practical circumstances and varying recommendations from public health and professional organizations. The collective experiences of SRN facilities may help (1) flag potential challenges for others to help them anticipate decision points, (2) them affirm their current practices in similar circumstances, and (3) identify areas for improved coordination from the international to local level for this pandemic and future outbreaks of emerging pathogens.

### Approaches unique to the COVID-19 pandemic

The SRN facilities identified and employed numerous strategies unique to the pandemic to maintain safety standards, including the following: extended use of disposable respirators beyond 1 day; respirators worn in combination with masks (surgical, cloth) to preserve them; storage of disposable respirators by the user in a paper bag between uses; reprocessing of disposable respirators via hydrogen peroxide vapor, ethylene oxide, UV irradiation, or moist heat treatments; self-production of PPE; self-production of test kits and testing materials; and extended use of disposable gowns.

The survey did not assess the time and resources that were required to conceive of, vet, obtain materials for, and implement these new approaches to optimizing healthcare supplies. However, the burden of doing this across settings, types of procedures, patients, healthcare roles, and local circumstances represents substantial opportunity cost at a time when healthcare facilities were under strain to prepare for, mitigate, and respond to the pandemic. These challenges also include implementing unfamiliar practices for HCP, such as long durations of extended use of disposable respirators and gowns. Additional opportunity costs may be inferred based on the resources dedicated by healthcare agencies such as the CDC to rapidly issue guidance to facilities struggling with supply limitations, and based on responses by professional organizations speaking on behalf of their memberships through guidelines, statements, and advocacy efforts.

### Future considerations and research needs

Local, national, and global communities face unknowns, among them whether SARS-CoV-2 triggers lasting neutralizing antibodies that prevent subsequent illness, the feasibility and timing of a COVID-19 vaccine, the effects of seasonal changes, and the impact of reopening different sectors of society. The COVID-19 pandemic has compelled institutions to take rapid, often practical actions that would benefit from further study and evaluation for safety, effectiveness, and cost. Several areas of research and evaluation are needed to assess what has been done so far and to find ways to strengthen facilities and their communities to prepare for the potential for new waves of COVID-19 cases and future outbreaks of emerging pathogens.

Approximately two-thirds of survey participants have received guidance from their institutions regarding potential therapies for COVID-19, PPE optimization strategies, patient triage, and visitor policies. However, only about one-third of survey participants received guidance from states and professional societies in these areas. These findings suggest the potential for these entities to take into account a broader view of the dilemmas faced by the healthcare community as well as the potential role for multidisciplinary considerations when developing and disseminating guidance. Perhaps further supporting the suggestion for better collaboration among multidisciplinary groups, the survey results revealed that PPE optimization was the most frequent topic covered in ethical guidance issued by institutions, states, and professional organizations. At the same time, it was the second most frequent topic on which facilities had sought guidance. Thus, more groups making recommendations on a critical topic may not lead to more clarity for facilities.

The impact of visitor policies enacted during the pandemic present additional areas for research. Although most SRN facilities allowed visitors for patients with suspected or confirmed COVID-19 disease at the end of life, as well as for minors and for birthing mothers, many reported limiting visitors to 1 per patient and required these visitors to wear PPE. The 2015 SHEA expert guidance “Isolation Precautions for Visitors” cites studies that have implicated visitors in hospital outbreaks, but it also notes the potential for negative psychosocial impact on patients and families from isolation practices.^11^ Research is needed to understand how patient outcomes have been affected by the isolation strategies put in place during the pandemic.

Many of the practices noted in this survey affect the amount of supplies available to the facility. Most facilities reported universal HCP respirator use in certain locations. Approximately half of respondents also indicated universal surgical masking for healthcare personnel facility-wide. The effectiveness of these strategies should be assessed for future planning. In addition, a number of facilities mentioned allowing HCP to use respirators during certain procedures on non–COVID-19 patients, even when not recommended. Research needs to be done on the impact of this practice on PPE utilization and COVID-19 transmission. In addition, research should examine the cost–benefit and safety of the various contingency strategies rapidly enacted during the pandemic due to sudden resource constraints (eg, extending the use of respirators beyond 1 day, storing respirators in paper bags, and reprocessing respirators). Finally, more information is needed regarding the utility of testing asymptomatic individuals prior to procedures or prior to discharge to skilled nursing facilities or hemodialysis units to better understand the impact of this type of testing on case detection and care for non–COVID-19 health conditions.

The study has several limitations. Although the survey addressed policies for testing of hospitalized versus nonhospitalized patients, other questions did not address inpatient versus outpatient settings. The survey did not differentiate between types of disposable respirators, and it applied the terms “reuse” and “extended use” to practices novel to the optimization of PPE during the pandemic (eg, extended use for a full day, with use again after storage in a paper bag). The ability to define a specific set of AGPs currently remains elusive^12–14^; thus, the survey and manuscript do not provide a grouping of AGPs.

Although it appears that the implementation of many of these policies was not driven by COVID-19 prevalence based on facilities’ reported test findings correlated to community prevalence, this finding should be interpreted with caution. Additionally, the survey did not collect information regarding whether facilities experienced a surge of patients. Comparisons of facilities further apart in prevalence would produce more dramatic effect estimates; however, all of the estimates are for a single “step up” in COVID-19 prevalence (eg, going from 6%–15% to 16%–25%). The survey response of 69 individual SRN facilities is not powered to reveal any particular magnitude of effect but rather to gather information about current practices. Practices differ based on where a particular community is on the epidemic curve, which can change rapidly.

## Supplementary material

For supplementary material accompanying this paper visit http://dx.doi.org/10.1017/ice.2020.303. click here to view supplementary material
