# Supplementary material for: Policies and practices of SHEA Research Network hospitals during the COVID-19 pandemic
Source: Infect Control Hosp Epidemiol. 2020 Jun 23:1–9. doi: 10.1017/ice.2020.303 (PMC7360941; doi:10.1017/ice.2020.303)
Supplement: Supplementary file 1 [file S0899823X20003037sup.zip › S0899823X20003037supp001.pdf]

(Captured by Internet Archive: Wayback Machine)

CDC Criteria for Return to Work for Healthcare Personnel with Confirmed or Suspected COVID-19 (Interim Guidance)

https://www.cdc.gov/coronavirus/2019-ncov/hcp/return-to-work.html

April 11, 2020 (day of SRN survey launch)

INTERNET ARCHIVE WaybackMachine
https://www.cdc.gov/coronavirus/2019-ncov/hcp/return-to-work.html Go
292 captures
4 Apr 2020 - 31 May 2020
MAR APR MAY
11
2019 2020 2021

Get Email Updates

To receive email updates about COVID-19, enter your email address:

Who this is for: Occupational health programs and public health officials making decisions about return to work for healthcare personnel (HCP) with confirmed COVID-19, or who have suspected COVID-19 (e.g., developed symptoms of a respiratory infection [e.g., cough, sore throat, shortness of breath, fever] but did not get tested for COVID-19).

Decisions about return to work for HCP with confirmed or suspected COVID-19 should be made in the context of local circumstances. Options include a test-based strategy or a non-test-based strategy (i.e., time-since-illness-onset and time-since-recovery strategy).

On This Page

Return to Work Criteria for HCP with Confirmed or Suspected COVID-19

Return to Work Practices and Work Restrictions

Crisis Strategies to Mitigate Staffing Shortages

Return to Work Criteria for HCP with Confirmed or Suspected COVID-19

Use one of the below strategies to determine when HCP may return to work in healthcare settings

1. *Test-based strategy.* Exclude from work until
   - Resolution of fever without the use of fever-reducing medications **and**
   - Improvement in respiratory symptoms (e.g., cough, shortness of breath), **and**
   - Negative results of an FDA Emergency Use Authorized molecular assay for COVID-19 from at least two consecutive nasopharyngeal swab specimens collected ≥24 hours apart (total of two negative specimens)[1]. See Interim Guidelines for Collecting, Handling, and Testing Clinical Specimens for 2019 Novel Coronavirus (2019-nCoV).
2. *Non-test-based strategy.* Exclude from work until
   - At least 3 days (72 hours) have passed *since recovery* defined as resolution of fever without the use of fever-reducing medications **and** improvement in respiratory symptoms (e.g., cough, shortness of breath); **and,**
   - At least 7 days have passed *since symptoms first appeared*

If HCP were never tested for COVID-19 but have an alternate diagnosis (e.g., tested positive for influenza), criteria for return to work should be based on that diagnosis.

April 15, 2020 (after April 13, 2020 CDC update)

INTERNET ARCHIVE WaybackMachine
https://www.cdc.gov/coronavirus/2019-ncov/hcp/return-to-work.html Go
MAR APR MAY
15
2019 2020 2021
292 captures
4 Apr 2020 - 31 May 2020

Get Email Updates

To receive email updates about COVID-19, enter your email address:

Email Address

What's this? Submit

Criteria for Return to Work for Healthcare Personnel with Confirmed or Suspected COVID-19 (Interim Guidance)

Summary of Recent Changes as of April 13, 2020

- Indicates a preference for use of the Test-based strategy to determine when HCP may return to work in healthcare settings
- Adds return to work criteria for HCP with laboratory-confirmed COVID-19 who have not had any symptoms
- Aligns with recommendations for universal source control for everyone in a healthcare facility during the pandemic.

CDC guidance for COVID-19 may be adapted by state and local health departments to respond to rapidly changing local circumstances.

Who this is for: Occupational health programs and public health officials making decisions about return to work for healthcare personnel (HCP) with confirmed COVID-19, or who have suspected COVID-19 (e.g., developed symptoms of a respiratory infection [e.g., cough, sore throat, shortness of breath, fever] but did not get tested for COVID-19).

Decisions about return to work for HCP with confirmed or suspected COVID-19 should be made in the context of local circumstances. Options include a test-based strategy or a non-test-based strategy (i.e., time-since-illness-onset and time-since-recovery strategy).

On This Page

Return to Work Criteria for HCP with Confirmed or Suspected COVID-19

Return to Work Practices and Work Restrictions

Strategies to Mitigate Healthcare Personnel Staffing Shortages

Return to Work Criteria for HCP with Confirmed or Suspected COVID-19

Use the *Test-based strategy* as the preferred method for determining when HCP may return to work in healthcare settings:

1. *Test-based strategy*. Exclude from work until
   - Resolution of fever without the use of fever-reducing medications **and**
   - Improvement in respiratory symptoms (e.g., cough, shortness of breath), **and**
   - Negative results of an FDA Emergency Use Authorized molecular assay for COVID-19 from at least two consecutive nasopharyngeal swab specimens collected ≥24 hours apart (total of two negative specimens) [1]. See Interim Guidelines for Collecting, Handling, and Testing Clinical Specimens for 2019 Novel Coronavirus (2019-nCoV).

If the *Test-based strategy* cannot be used, the *Non-test-based strategy* may be used for determining when HCP may return to work in healthcare settings:

2. Non-test-based strategy. Exclude from work until
   - At least 3 days (72 hours) have passed *since recovery* defined as resolution of fever without the use of fever-reducing medications **and** improvement in respiratory symptoms (e.g., cough, shortness of breath); **and,**
   - At least 7 days have passed *since symptoms first appeared*

HCP with laboratory-confirmed COVID-19 who have not had any symptoms should be excluded from work until 10 days have passed since the date of their first positive COVID-19 diagnostic test assuming they have not subsequently developed symptoms since their positive test.

If HCP had COVID-19 ruled out and have an alternate diagnosis (e.g., tested positive for influenza), criteria for return to work should be based on that diagnosis.

[illegible]

CDC Symptoms of Coronavirus

https://www.cdc.gov/coronavirus/2019-ncov/symptoms-testing/symptoms.html

April 14, 2020

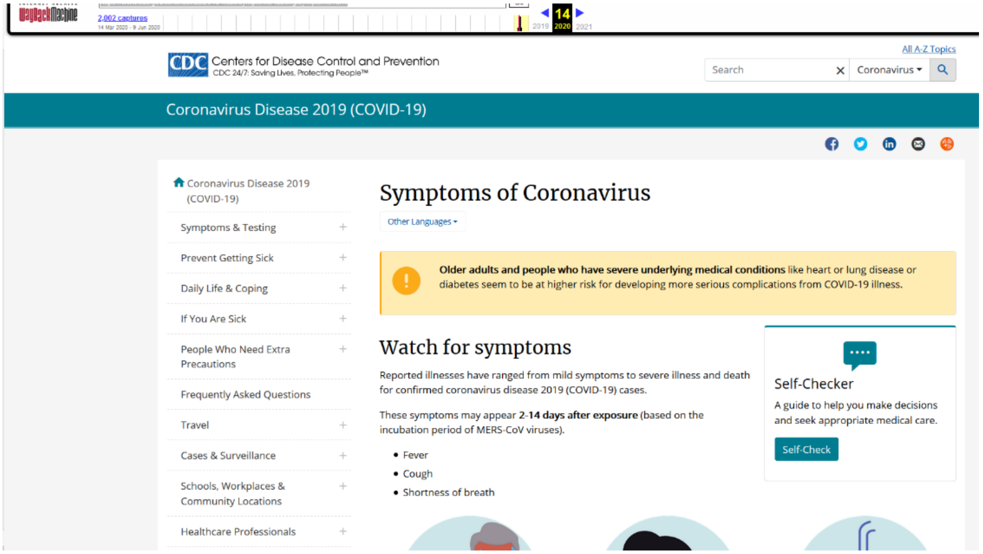

2,002 captures

14 Mar 2020 - 9 Jun 2020

14 2019 2020 2021

All A-Z Topics

CDC Centers for Disease Control and Prevention

CDC 24/7: Saving Lives, Protecting People™

Search Coronavirus

Coronavirus Disease 2019 (COVID-19)

Coronavirus Disease 2019 (COVID-19)

Symptoms & Testing

Prevent Getting Sick

Daily Life & Coping

If You Are Sick

People Who Need Extra Precautions

Frequently Asked Questions

Travel

Cases & Surveillance

Schools, Workplaces & Community Locations

Healthcare Professionals

Symptoms of Coronavirus

Other Languages

Older adults and people who have severe underlying medical conditions like heart or lung disease or diabetes seem to be at higher risk for developing more serious complications from COVID-19 illness.

Watch for symptoms

Reported illnesses have ranged from mild symptoms to severe illness and death for confirmed coronavirus disease 2019 (COVID-19) cases.

These symptoms may appear **2-14 days after exposure** (based on the incubation period of MERS-CoV viruses).

- Fever
- Cough
- Shortness of breath

Self-Checker

A guide to help you make decisions and seek appropriate medical care.

Self-Check

April 17, 2020 (list updated, remains same through survey close)

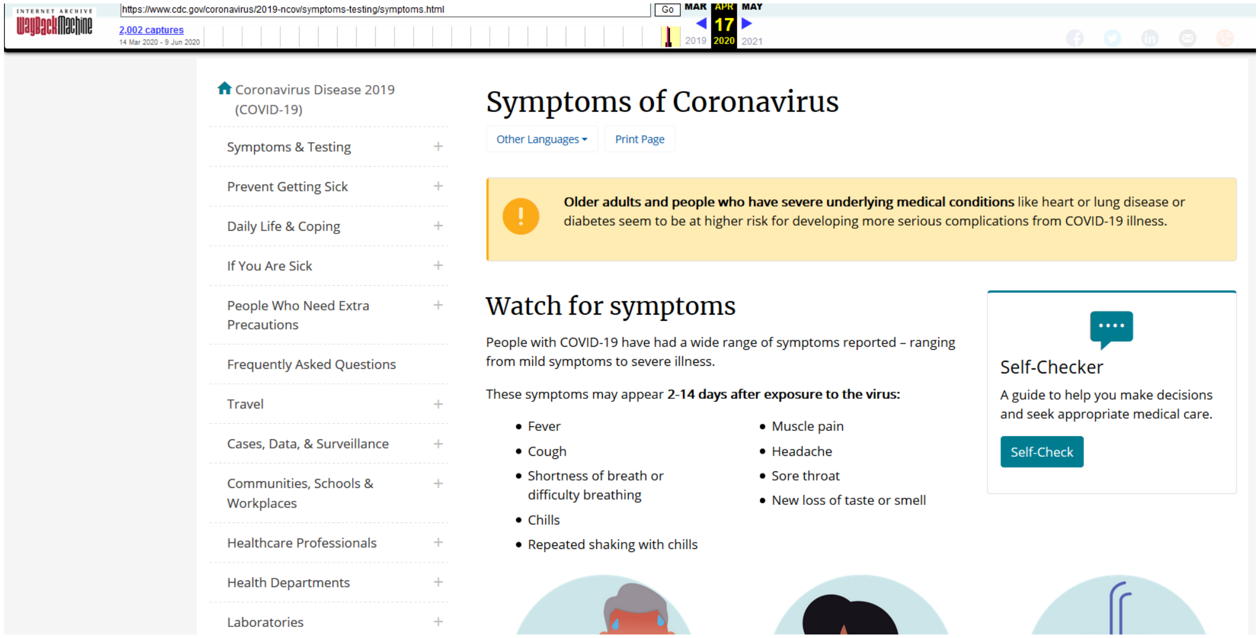

https://www.cdc.gov/coronavirus/2019-ncov/symptoms-testing/symptoms.html Go

MAR APR MAY

17

2019 2020 2021

2,002 captures

14 Mar 2020 - 9 Jun 2020

Coronavirus Disease 2019 (COVID-19)

Symptoms & Testing

Prevent Getting Sick

Daily Life & Coping

If You Are Sick

People Who Need Extra Precautions

Frequently Asked Questions

Travel

Cases, Data, & Surveillance

Communities, Schools & Workplaces

Healthcare Professionals

Health Departments

Laboratories

Symptoms of Coronavirus

Other Languages Print Page

Older adults and people who have severe underlying medical conditions like heart or lung disease or diabetes seem to be at higher risk for developing more serious complications from COVID-19 illness.

Watch for symptoms

People with COVID-19 have had a wide range of symptoms reported – ranging from mild symptoms to severe illness.

These symptoms may appear **2-14 days after exposure to the virus:**

- Fever
- Cough
- Shortness of breath or difficulty breathing
- Chills
- Repeated shaking with chills
- Muscle pain
- Headache
- Sore throat
- New loss of taste or smell

Self-Checker

A guide to help you make decisions and seek appropriate medical care.

Self-Check

CDC Evaluating and Testing Persons for Coronavirus Disease 2019 (COVID-19)

https://www.cdc.gov/coronavirus/2019-nCoV/hcp/clinical-criteria.html

April 15, 2020 (no change during survey duration, April 11-23, 2020)

INTERNET ARCHIVE WaybackMachine https://www.cdc.gov/coronavirus/2019-nCoV/hcp/clinical-criteria.html Go
1,887 captures
29 Jan 2020 - 10 Jun 2020
MAR APR MAY 15 2019 2020 2021

Healthcare providers should **immediately** notify their local or state health department in the event of the identification of a PUI for COVID-19. When working with your local or state health department check their available hours.

[illegible]ive email updates [illegible]ut COVID-19, enter your email address:

Email Address

What's this? Submit

Criteria to Guide Evaluation and Laboratory Testing for COVID-19

Clinicians considering testing of persons with possible COVID-19 should continue to work with their local and state health departments to coordinate testing through public health laboratories, or use COVID-19 diagnostic testing, authorized by the Food and Drug Administration under an Emergency Use Authorization (EUA) through clinical laboratories. Increasing testing capacity will allow clinicians to consider COVID-19 testing for a wider group of symptomatic patients.

Clinicians should use their judgment to determine if a patient has signs and symptoms compatible with COVID-19 and whether the patient should be tested. Most patients with confirmed COVID-19 have developed fever[1] and/or symptoms of acute respiratory illness (e.g., cough, difficulty breathing). Priorities for testing include:

Other considerations that may guide testing are epidemiologic factors such as the occurrence of local community transmission of COVID-19 infections in a jurisdiction. Clinicians are strongly encouraged to test for other causes of respiratory illness.

Priorities for testing patients with suspected COVID-19 infection

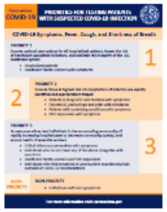

[PDF - 202 KB]

PRIORITY 1
Ensure optimal care options for all hospitalized patients, lessen the risk of nosocomial infections, and maintain the integrity of the healthcare system

- Hospitalized patients
- Symptomatic healthcare workers

PRIORITY 2
Ensure that those who are at highest risk of complication of infection are rapidly identified and appropriately triaged

- Patients in long-term care facilities with symptoms
- Patients 65 years of age and older with symptoms
- Patients with underlying conditions with symptoms
- First responders with symptoms

PRIORITY 3
As resources allow, test individuals in the surrounding community of rapidly increasing hospital cases to decrease community spread, and ensure health of essential workers

- Critical infrastructure workers with symptoms
- Individuals who do not meet any of the above categories with symptoms
- Health care workers and first responders
- Individuals with mild symptoms in communities experiencing high COVID-19 hospitalizations

NON-PRIORITY
- Individuals without symptoms
