# Supplementary material for: Policies and practices of SHEA Research Network hospitals during the COVID-19 pandemic
Source: Infect Control Hosp Epidemiol. 2020 Jun 23:1–9. doi: 10.1017/ice.2020.303 (PMC7360941; doi:10.1017/ice.2020.303)
Supplement: Supplementary file 1 [file S0899823X20003037sup.zip › S0899823X20003037supp002.docx]

# **VI. Appendix 1: “Current COVID-19 Practices” Survey**

1) SRN ID: **_________________________________________________**

2) State/Province, Country: **_______________________________________________**

3) For patients tested in your facility, how many are COVID+?

( ) <5%

( ) 6-15%

( ) 16-25%

( ) >25%

4) What is the status of PPE supplies in your facility?

|  | **Sustainable for pandemic** | **Adequate for current situation** | **Limited, expecting improvements** | **Limited, expecting declines** | **Crisis-level (almost out/none)** |
| --- | --- | --- | --- | --- | --- |
| Respirators | [ ] | [ ] | [ ] | [ ] | [ ] |
| Surgical masks | [ ] | [ ] | [ ] | [ ] | [ ] |
| Gowns | [ ] | [ ] | [ ] | [ ] | [ ] |
| Gloves | [ ] | [ ] | [ ] | [ ] | [ ] |
| Eye covering | [ ] | [ ] | [ ] | [ ] | [ ] |

5) Which PPE strategies is your facility using? Check all that apply.

|  | **Certain units (e.g. COVID-19, ICU, ER)** | **All clinical care areas (including ambulatory areas)** | **Entire facility** | **Currently using** | **Planning to use** | **Not using/planning to use** |
| --- | --- | --- | --- | --- | --- | --- |
| Universal HCP respirator use | [ ] | [ ] | [ ] | [ ] | [ ] | [ ] |
| Universal HCP surgical masking | [ ] | [ ] | [ ] | [ ] | [ ] | [ ] |
| Respirator re-use, same HCP for 1 day | [ ] | [ ] | [ ] | [ ] | [ ] | [ ] |
| Respirator re-use, same HCP for >1 day | [ ] | [ ] | [ ] | [ ] | [ ] | [ ] |
| Respirator re-use, same HCP, storage in paper bag | [ ] | [ ] | [ ] | [ ] | [ ] | [ ] |
| Respirator re-use, same HCP, worn with new surgical mask | [ ] | [ ] | [ ] | [ ] | [ ] | [ ] |
| Respirator re-use, same HCP, worn with laundered cloth mask | [ ] | [ ] | [ ] | [ ] | [ ] | [ ] |
| Respirator reprocessing - hydrogen vapor | [ ] | [ ] | [ ] | [ ] | [ ] | [ ] |
| Respirator reprocessing - EtO | [ ] | [ ] | [ ] | [ ] | [ ] | [ ] |
| Respirator reprocessing - UV | [ ] | [ ] | [ ] | [ ] | [ ] | [ ] |
| Coverall re-use | [ ] | [ ] | [ ] | [ ] | [ ] | [ ] |
| Gown re-use | [ ] | [ ] | [ ] | [ ] | [ ] | [ ] |

6) What does your facility recommend* for HCP who are involved in procedures performed on suspected or confirmed COVID-19 patients? Check all that apply.
*based on actual circumstances/supplies in your facility

|  | **N95** | **PAPR** | **Half-mask respirator** | **Surgical mask** | **Eyewear (shield, goggles)** | **Respirator allowed, but not recommended*** |
| --- | --- | --- | --- | --- | --- | --- |
| NP swab (done in room) | [ ] | [ ] | [ ] | [ ] | [ ] | [ ] |
| NP swab (done outdoors, e.g. employee screening) | [ ] | [ ] | [ ] | [ ] | [ ] | [ ] |
| Intubation | [ ] | [ ] | [ ] | [ ] | [ ] | [ ] |
| Extubation | [ ] | [ ] | [ ] | [ ] | [ ] | [ ] |
| Bag masking | [ ] | [ ] | [ ] | [ ] | [ ] | [ ] |
| Bronchoscopy | [ ] | [ ] | [ ] | [ ] | [ ] | [ ] |
| ENT scope | [ ] | [ ] | [ ] | [ ] | [ ] | [ ] |
| ENT surgery | [ ] | [ ] | [ ] | [ ] | [ ] | [ ] |
| Medication administration via continuous nebulizer | [ ] | [ ] | [ ] | [ ] | [ ] | [ ] |
| High-frequency oscillating ventilation (HFOV) | [ ] | [ ] | [ ] | [ ] | [ ] | [ ] |
| High-flow nasal oxygen (HFNO) (high-flow nasal cannula) with humidication | [ ] | [ ] | [ ] | [ ] | [ ] | [ ] |
| HFNO without humidication | [ ] | [ ] | [ ] | [ ] | [ ] | [ ] |
| Chest tube placement | [ ] | [ ] | [ ] | [ ] | [ ] | [ ] |
| Presence of tracheostomy | [ ] | [ ] | [ ] | [ ] | [ ] | [ ] |
| Induction of sputum | [ ] | [ ] | [ ] | [ ] | [ ] | [ ] |
| Chest compressions | [ ] | [ ] | [ ] | [ ] | [ ] | [ ] |
| Use of secretion clearing devices | [ ] | [ ] | [ ] | [ ] | [ ] | [ ] |
| When ventilator circuits are broken | [ ] | [ ] | [ ] | [ ] | [ ] | [ ] |
| Non-invasive ventilation (NIV), e.g. BiPAP, CPAP | [ ] | [ ] | [ ] | [ ] | [ ] | [ ] |
| Video-assisted thoracoscopic surgery (VATS) | [ ] | [ ] | [ ] | [ ] | [ ] | [ ] |
| CABG with internal mammary artery | [ ] | [ ] | [ ] | [ ] | [ ] | [ ] |
| CABG with redo sternotomy | [ ] | [ ] | [ ] | [ ] | [ ] | [ ] |
| C-section | [ ] | [ ] | [ ] | [ ] | [ ] | [ ] |
| Second stage of labor | [ ] | [ ] | [ ] | [ ] | [ ] | [ ] |
| Non-surgical delivery | [ ] | [ ] | [ ] | [ ] | [ ] | [ ] |
| Care of preterm babies in isolates with NIV (NCPAP, NIPPV, NHFO, HFNC) | [ ] | [ ] | [ ] | [ ] | [ ] | [ ] |
| Electrocautery of blood | [ ] | [ ] | [ ] | [ ] | [ ] | [ ] |
| Electrocautery of GI tissue | [ ] | [ ] | [ ] | [ ] | [ ] | [ ] |
| Electrocautery of other body fluids | [ ] | [ ] | [ ] | [ ] | [ ] | [ ] |
| Neurosurgery | [ ] | [ ] | [ ] | [ ] | [ ] | [ ] |
| Laparoscopy | [ ] | [ ] | [ ] | [ ] | [ ] | [ ] |
| Endoscopy | [ ] | [ ] | [ ] | [ ] | [ ] | [ ] |

7) Exceptions/other comments: **____________________________________________**

8) Does your facility recommend* that HCP who are involved in procedures on patients NOT suspected for COVID-19 infection wear PPE that is different than standard precautions?
*based on actual circumstances/supplies in your facility

( ) Yes, for certain procedures ( ) No, we are following standard precautions

9) What does your facility recommend* for HCP who are involved in procedures on patients NOT suspected for COVID-19 infection that is different than standard precautions? Check all that apply.
*based on actual circumstances/supplies in your facility

|  | **N95** | **PAPR** | **Half-mask respirator** | **Surgical mask** | **Eyewear (shield, goggles)** | **Respirator allowed, but not recommended*** |
| --- | --- | --- | --- | --- | --- | --- |
| NP swab (done in room) | [ ] | [ ] | [ ] | [ ] | [ ] | [ ] |
| NP swab (done outdoors, e.g. employee screening) | [ ] | [ ] | [ ] | [ ] | [ ] | [ ] |
| Intubation | [ ] | [ ] | [ ] | [ ] | [ ] | [ ] |
| Extubation | [ ] | [ ] | [ ] | [ ] | [ ] | [ ] |
| Bag masking | [ ] | [ ] | [ ] | [ ] | [ ] | [ ] |
| Bronchoscopy | [ ] | [ ] | [ ] | [ ] | [ ] | [ ] |
| ENT scope | [ ] | [ ] | [ ] | [ ] | [ ] | [ ] |
| ENT surgery | [ ] | [ ] | [ ] | [ ] | [ ] | [ ] |
| Medication administration via continuous nebulizer | [ ] | [ ] | [ ] | [ ] | [ ] | [ ] |
| High-frequency oscillating ventilation (HFOV) | [ ] | [ ] | [ ] | [ ] | [ ] | [ ] |
| High-flow nasal oxygen (HFNO) (high-flow nasal cannula) with humidication | [ ] | [ ] | [ ] | [ ] | [ ] | [ ] |
| HFNO without humidication | [ ] | [ ] | [ ] | [ ] | [ ] | [ ] |
| Chest tube placement | [ ] | [ ] | [ ] | [ ] | [ ] | [ ] |
| Presence of tracheostomy | [ ] | [ ] | [ ] | [ ] | [ ] | [ ] |
| Induction of sputum | [ ] | [ ] | [ ] | [ ] | [ ] | [ ] |
| Chest compressions | [ ] | [ ] | [ ] | [ ] | [ ] | [ ] |
| Use of secretion clearing devices | [ ] | [ ] | [ ] | [ ] | [ ] | [ ] |
| When ventilator circuits are broken | [ ] | [ ] | [ ] | [ ] | [ ] | [ ] |
| Non-invasive ventilation (NIV), e.g. BiPAP, CPAP | [ ] | [ ] | [ ] | [ ] | [ ] | [ ] |
| Video-assisted thoracoscopic surgery (VATS) | [ ] | [ ] | [ ] | [ ] | [ ] | [ ] |
| CABG with internal mammary artery | [ ] | [ ] | [ ] | [ ] | [ ] | [ ] |
| CABG with redo sternotomy | [ ] | [ ] | [ ] | [ ] | [ ] | [ ] |
| C-section | [ ] | [ ] | [ ] | [ ] | [ ] | [ ] |
| Second stage of labor | [ ] | [ ] | [ ] | [ ] | [ ] | [ ] |
| Non-surgical delivery | [ ] | [ ] | [ ] | [ ] | [ ] | [ ] |
| Care of preterm babies in isolettes with NIV (NCPAP, NIPPV, NHFO, HFNC) | [ ] | [ ] | [ ] | [ ] | [ ] | [ ] |
| Electrocautery of blood | [ ] | [ ] | [ ] | [ ] | [ ] | [ ] |
| Electrocautery of GI tissue | [ ] | [ ] | [ ] | [ ] | [ ] | [ ] |
| Electrocautery of other body fluids | [ ] | [ ] | [ ] | [ ] | [ ] | [ ] |
| Neurosurgery | [ ] | [ ] | [ ] | [ ] | [ ] | [ ] |
| Laparoscopy | [ ] | [ ] | [ ] | [ ] | [ ] | [ ] |
| Endoscopy | [ ] | [ ] | [ ] | [ ] | [ ] | [ ] |

10) Exceptions/other comments: **____________________________________________**

11) Do you have in-house testing?

( ) Yes ( ) No

12) What is the turn-around time for test results? **_________________________________________________**

13) Which types of patients is your facility testing for COVID-19 infection? Check all that apply.

|  | **Hospitalized** | **Not hospitalized** |
| --- | --- | --- |
| Patients with respiratory symptoms | [ ] | [ ] |
| Isolated fever | [ ] | [ ] |
| Patients with GI symptoms | [ ] | [ ] |
| Asymptomatic patients undergoing certain procedures (please specify below) | [ ] | [ ] |
| Asymptomatic patients being discharged to a hemodialysis unit | [ ] | [ ] |
| Asymptomatic patients being discharged to nursing home, SNF, or LTACH | [ ] | [ ] |

14) Please list procedures for which you are testing asymptomatic patients: ______________________

15) Is your facility using HCP return-to-work crisis-level [mitigation strategies](https://www.cdc.gov/coronavirus/2019-ncov/hcp/return-to-work.html?CDC_AA_refVal=https%3A%2F%2Fwww.cdc.gov%2Fcoronavirus%2F2019-ncov%2Fhealthcare-facilities%2Fhcp-return-work.html) for staffing shortages? (CDC link opens in new window)

[ ] No, we are following test-based return-to-work criteria (CDC)

[ ] No, we are following non-test-based return to work criteria (CDC)

[ ] Yes, with evaluation by occupational health

[ ] Yes, with certain restrictions: _________________________________________________

16) Due to shortages, is your facility self-producing:

[ ] Viral transport media

[ ] Viral collection swabs

[ ] Collection tubes

[ ] Other: _________________________________________________

17) When serology testing becomes available, will your facility consider a positive COVID-19 IgG indicative of immunity?

( ) Yes

( ) No

( ) Don't know

( ) Other: _________________________________________________

18) Does your facility allow visitors of suspected or confirmed COVID-19 patients?

( ) Yes

( ) Certain circumstances (e.g. end-of-life)

( ) No

19) How many visitors does your facility allow? If no visitors are allowed, leave as is.

End-of-life visits: _________________________________________________

Birthing partners: _________________________________________________

Pediatric patients: _________________________________________________

Other: _________________________________________________

20) If allowed, does your facility require that visitor(s) wear PPE during:

[ ] End-of-life visits

[ ] Childbirth

[ ] Pediatric visits

[ ] N/A (my facility doesn't allow visitors for COVID-19 patients)

[ ] Other: _________________________________________________

21) For HCP, is your facility doing:

|  | **Certain units (e.g. COVID-19, ICU, ER)** | **All clinical care areas (including ambulatory areas)** | **Entire facility** | **Voluntary** | **Required** | **Not using/planning to use** |
| --- | --- | --- | --- | --- | --- | --- |
| Once daily symptom assessment | [ ] | [ ] | [ ] | [ ] | [ ] | [ ] |
| Twice daily symptom assessment | [ ] | [ ] | [ ] | [ ] | [ ] | [ ] |
| Daily testing for COVID-19 | [ ] | [ ] | [ ] | [ ] | [ ] | [ ] |
| Patients wear masks (e.g. cloth, surgical) before HCP enter room | [ ] | [ ] | [ ] | [ ] | [ ] | [ ] |
| Facility-provided scrubs | [ ] | [ ] | [ ] | [ ] | [ ] | [ ] |
| Facility-provided hair covers/bouffant caps | [ ] | [ ] | [ ] | [ ] | [ ] | [ ] |
| Facility laundering of scrubs at the end of shift | [ ] | [ ] | [ ] | [ ] | [ ] | [ ] |
| Onsite shower for HCP at end of shift | [ ] | [ ] | [ ] | [ ] | [ ] | [ ] |
| Onsite/local accommodations during pandemic | [ ] | [ ] | [ ] | [ ] | [ ] | [ ] |
| Contact tracing for COVID+ HCP | [ ] | [ ] | [ ] | [ ] | [ ] | [ ] |

22) Have you received guidance with respect to ethical considerations for (check all that apply):

|  | **Institution** | **State** | **Professional society** | **No, but seeking guidance** | **Have not sought guidance** |
| --- | --- | --- | --- | --- | --- |
| Potential COVID-19 therapeutics | [ ] | [ ] | [ ] | [ ] | [ ] |
| PPE conservation strategies | [ ] | [ ] | [ ] | [ ] | [ ] |
| Patient triage | [ ] | [ ] | [ ] | [ ] | [ ] |
| Equipment modifications | [ ] | [ ] | [ ] | [ ] | [ ] |
| Visitor policies | [ ] | [ ] | [ ] | [ ] | [ ] |

23) Comments: **____________________________________________**
